# Identification of End-Binding 1 Protein as Novel α-4 Giardin-Binding Partners in *Giardia lamblia* Trophozoites

**DOI:** 10.1007/s11686-023-00774-y

**Published:** 2024-01-11

**Authors:** Kaiyue Zhang, Hai’e Shen, Yi Wang, Hailin Shen, Chenshuo Zhang, Xu Zou, Yuan Yu, Xifeng Tian, Yang Wang

**Affiliations:** 1 College of Life Sciences, North China University of Science and Technology, Tangshan, 063000 China; 2 Department of Clinical Laboratory, North China University of Science and Technology Affiliated Hospital, Tangshan, 063000 China; 3 The Second Hospital of Tangshan, Tangshan, 063000 China; 4 College of Basic Medical Sciences, North China University of Science and Technology, Tangshan, 063000 China

**Keywords:** *Giardia lamblia*, α-4 Giardin, End-binding 1 protein, Yeast two hybridization, Ribosomal protein L21

## Abstract

**Background:**

*Giardia lamblia* (syn. *G. intestinalis*, *G. duodenalis*) is a primitive opportunistic protozoon, and one of the earliest differentiated eukaryotes. Despite its primitive nature, *G. lamblia* has a sophisticated cytoskeleton system, which is closely related to its proliferation and pathogenicity. Meanwhile, α giardin is a *G. lamblia*-specific cytoskeleton protein, which belongs to the annexin superfamily. Interestingly, *G. lamblia* has 21 annexin-like α giardins, i.e., more than higher eukaryotes. The functional differences among α giardin members are not fully understood.

**Methods:**

We took α-4 giardin, a member of α giardin family, as a research object. A morpholino-mediated knockdown experiment was performed to identify the effect of α-4 giardin on *G. lamblia* trophozoites biological traits. A yeast two-hybrid cDNA library of *G. lamblia* strain C2 trophozoites was screened for interaction partners of α-4 giardin. Co-immunoprecipitation and fluorescent colocalization confirmed the relationship between *G. lamblia* EB1 (gEB1) and α-4 giardin.

**Results:**

α-4 Giardin could inhibit the proliferation and adhesion of *G. lamblia* trophozoites. In addition, it interacted with *G. lamblia* EB1 (gEB1).

**Conclusions:**

α-4 Giardin was involved in proliferation and adhesion in *G. lamblia* trophozoites, and EB1, a crucial roles in mitosis, was an interacting partner of α-4 giardin.

## Introduction

*Giardia lamblia* (syn. *G. intestinalis*, *G. duodenalis*), a common, opportunistic intestinal parasitic protozoa with worldwide distribution, can parasitize the small intestine of humans and many other mammals, causing giardiasis that mainly manifests as diarrhea, abdominal pain, nausea and malabsorption symptoms. The life cycle of *G. lamblia* includes the infective, immotile cyst and the vegetative, motile trophozoite. Cysts are resistant forms and responsible for the transmission of giardiasis. After cysts pass through the stomach, they are transformed into trophozoites. *G. lamblia* trophozoites attach strongly to intestinal epithelial cells via a ventral adhesive disc, a concave cytoskeletal structure surrounded by the plasma membrane, and cause significant damage and disruption to the intestinal epithelium [1, 2].

As one of the earliest diverging eukaryotes, *G. lamblia* represents an excellent model system for assessing basic eukaryotic processes [3]. *G. lamblia* is only a primitive eukaryotic unicellular organism which has a highly developed and fine cytoskeleton system similar to that of higher eukaryotes, but the structures formed by these proteins in *G. lamblia* are unique. Microtubules, microfilaments, and related proteins forming the cytoskeleton mainly consist of four pairs of flagella, the adhesive disc, the median body and the funis [4, 5]. The adhesive disc and flagella are essential for trophozoite mobility, cell division and proliferation, as well as attachment to epithelial cells in the small intestinal mucosa [6, 7]. The parasitic *G. lamblia* trophozoites in the small intestine can cause diarrhea, which is mainly explained by the parasite relying on its adhesive disc to attach to the surface of small intestinal epithelial cells, assisted by flagella. As a result, the intestinal microvilli are damaged directly, which causes diffuse dysfunction of microvilli and local inflammation. Eventually, diarrhea occurs as a result of nutrient and water absorption disorder [8]. Therefore, the cytoskeleton plays a critical role in the pathogenesis of *G. lamblia* infection [9, 10].

It was reported that the cytoskeletal proteins of *G. lamblia* mainly include giardin, dynein, tubulin, actin and several related proteins [5]. Among them, giardin is a unique component of *G. lamblia* cytoskeleton. Crossley and Holberton first reported that giardin is a unique component of *G. lamblia* cytoskeleton in 1983. Giardin is divided into four types, including α, β, γ, and δ-giardin [11, 12]. Alpha-giardin is the largest family, with 21 variants with molecular weights ranging from 29 to 38 kDa. The α-giardin variants are named by Arabic numbers preceded by the Greek letter "α", with a "-" inserted between them. Alpha-giardin include α-1 to α-6, α-7.1, α-7.2, α-7.3, and α-8 to α-19 [13, 14]. The expression of α-giardin is strictly regulated, and there are significant differences in the levels of expression of its members [15] with distinct subcellular localizations, suggesting that each member may have different significance in the survival of *G. lamblia* [16]. Currently, studies assessing giardin’s functions are scarce.

Alpha-4 giardin is a member of the α giardin family. In a previous study, we prepared a specific antibody against α-4 giardin and identified its localization in *G. lamblia* trophozoites [17]. However, the specific function of α-4 giardin remains unclear.

A given protein does not usually function alone, more or less interacting with other proteinaceous components. It is helpful to assess the functions and mechanisms of unknown proteins by recognizing protein components that interact with the latter. In this study, α-4 giardin was down-regulated by morpholinos for biological traits evaluation. A yeast two-hybrid cDNA library obtained from *G. lamblia* strain C2 trophozoites was screened for interaction partners of α-4 giardin. The interaction between the partner protein and α-4 giardin was confirmed by co-immunoprecipitation and fluorescent colocalization assays. The results provide novel insights into the mechanism of α-4 giardin’s biological function.

## Materials and Methods

### Parasite Strain

The *G. lamblia* isolate utilized in these studies (C2), which belongs to Genotype A, was derived from a patient in southwest China [18] Trophozoites were cultured and propagated in modified TYI-S-33 medium. Subcultures were performed every 3 days [19].

### Construction of α-4 Giardin Knockdown by Morpholinos.

Translation-blocking morpholinos was used to knockdown α-4 giardin in *G. lamblia* trophozoites. The 25-mers α-4 giardin morpholino (α-4 MO: 5ʹ- CACTGTGGATACCGTGGCAGACATT-3ʹ) and Control morpholino with five mismatched bases (C-MO:5ʹ -gACTGTcGAcACgGTGGCAGAgATT-3ʹ) were designed based on mRNA for α-4 giardin. Control morpholinos contain five mismatched bases disrupting the pairing of morpholinos with α-4 giardin mRNA (Lowercase letters indicate mispaired bases). For the negative control, a volume of sterile water equal to the volume of the morpholino suspension was added. The α-4 giardin morpholino, mispair control and water were transfected in log-phase trophozoites, respectively, via electroporation as previously described [20]. The transfected cells were transferred to fresh *G. lamblia* culture medium and incubated at 37℃.

### Effect of α-4 Giardin on Parasite Growth

To evaluate the effect of α-4 giardin on parasite growth, α-4 giardin morpholino transfected trophozoites were cultured with a density of 50,000 parasites/mL in TYI-S-33 medium at 37 ℃ for 24, 48 and 72 h. Mispair morpholino transfected and water transfected *G. lamblia* trophozoites were used as controls, respectively. The cells were harvested by cooling the culture tubes and the cells were counted using a Neubauer chamber.

### Effect of α-4 Giardin on Adherence

To evaluate the effect of α-4 giardin on adherence, an adherence inhibition assay was performed as previously described [21]. Briefly, 50,000 parasites/mL were grown at concentrations and time described above. After incubation, medium containing non-adherent cells was removed and kept on ice, tubes were filled with cold phosphate buffered saline (PBS) and placed on ice bath for 30 min to dislodge the adherent cells. The numbers of adherent and non-adherent trophozoites were determined by counting in a Neubauer chamber. The results were expressed as percentage of non-adhered trophozoites in relation to the total number of cells.

### Yeast Two-Hybrid Screen

A Matchmaker two-hybrid system (Clontech, Mountain View, USA) was used to screen proteins interacting with α-4 giardin from a yeast two-hybrid cDNA library of *G. lamblia* strain C2 trophozoites. The bait yeast strain Y187 transformed with pGBKT7-α-4 was cultured in 50 mL SD/-Trp liquid medium at 30 °C until an OD600 of 0.8. The bait yeast strain was collected by centrifugation for 5 min at 1000×*g* and resuspended in 5 mL SD/-Trp liquid medium, and combined with 1 mL of library strain in a 1-L sterile flask. Culture was performed in 45 mL 2 × YPDA (50 mg/mL kanamycin) liquid medium at 30 °C with shaking for 22 h. From a drop of the mated culture, fusion was analyzed by microscopy to assess the presence or absence of zygotes. When zygotes were observed, the mated cultures were collected and resuspended in 5 mL 0.5 × YPDA/kanamycin liquid medium, spread on selective media SD/–Leu/–Trp/–His plates, and cultured for 5 days at 30 °C. To reduce false positives, all positive colonies were transferred to new selective media SD/–Leu/–Trp/–His/-Ade and SD/–Leu/–Trp plates for further screening. Clones growing on both plates were inoculated on another SD/-Leu/-Trp/-His/-Ade plate for a second screening. All positive clones were picked and cultured in selective liquid medium (SD/–Leu/–Trp/–His/-Ade/kanamycin) overnight at 30 °C. To estimate the sizes of specific inserts from putative positive clones, PCR was carried out using universal primers for pGADT7. Based on the PCR results, prey plasmids from putative positive clones were extracted using the Easy Yeast Plasmid Isolation kit (Clontech, Mountain View, USA), transformed into E. coli DH5a competent cells, and cultured on LB/Amp plates for sequencing.

### Confirmation of Positive Clones of Interest

To eliminate false positives, the library plasmids were co-transferred into yeast cells with the pGBKT7/α-4 bait vector, and spread on a SD/–Leu/–Trp plate. The cultured clones were inoculated on SD/-Leu/-Trp and SD/-Leu/-Trp/-His/-Ade plates, respectively, to assess the interaction between the plasmid encoded protein and the bait protein. In addition, positive (pGBKT7-p53 and pGADT7-T) and negative (pGBKT7 and pGADT7) controls were assessed simultaneously. Clones were selected from each set of plates and inoculated into SD/-Leu/-Trp and SD/-Leu/-Trp/-His/-Ade plates, respectively, and observed for growth. Sequences of putative positive clones were analyzed using BLAST programs at NCBI (http://www.ncbi.nlm.nih.gov/BLAST/) and the *G. lamblia* genome sequence (http://giardiadb.org/giardiadb/).

### Plasmid Construction and Transient Transfection

The DNA fragment encoding α-4 giardin gene was amplified from the constructed α-4 giardin bait plasmid by primers (F: 5ʹ-CCCAAGCTTGCCACCATGGACTACAAAGACGATGACGATAAAATGTCTGCCACGGTATCCAC-3ʹ and R: 5ʹ-CGGAATTCCTACTCCACGCGCCAAAAGACTAG-3ʹ). The primers contained HindIII and EcoRI restriction sites (underlined) and a FLAG epitope tag coding sequence (in bold) was placed in the forward primer upstream the α-4 giardin coding sequence. The DNA fragment encoding gEB1 gene was amplified from *G. lamblia* cDNA by primers (F: 5ʹ-CGGAATTCATGCCGCCGGTAAAAGCACCCG-3ʹ and R: 5ʹ-GGGGTACCTTACTGATGATACTCCGCATACAGAATATC-3ʹ). The primers contained EcoRI and KpnI restriction sites (underlined). Amplified DNA fragment of α-4 giardin and gEB1 was inserted into pcDNA3.1(+) and pCMV-N-Myc by double digestion, respectively. The constructed plasmids were named as pcDNA3.1-FLAG-α-4 and pCMV-Myc-gEB1, respectively.

### Production of Anti-gEB1 and Anti-α-4 Giardin Antibodies

To produce antibodies specifically against the gEB1 protein, a synthesized antigenic peptide with the sequence AVTKTSKPGNRSGST which corresponds to residues 135–149 of gEB1 was conjugated with Keyhole Limpet Hemocyanin (KLH) and injected intraperitoneally into rabbits after mixed with Freund’s complete adjuvant, followed once weekly by 4 injections of incomplete Freund’s adjuvant. Seven days after the last injection, blood was extracted, and the serum was collected and purified by protein A sefinose column (Sangon, Shanghai, China). Anti-α-4 giardin monoclonal antibody was generated by immunizing mice with a synthesized antigenic peptide with the sequence VKHDRKYRKSIKSDAEAFR corresponding to residues 145–163 of α-4 giardin. The specificity of the antibodies were confirmed by Western blot using the lysate of trophozoites and stored in aliquots at -20℃ until use.

### Co-immunoprecipitation

Co-immunoprecipitation analysis was employed to verify the interaction between α-4 giardin and *G. lamblia* end-binding 1 protein (gEB1). Plasmids pcDNA3.1-FLAG -α-4 and pCMV-Myc-gEB1 were cotransfected into 293 T cells using Lipofectamine 2000 (Invitrogen, Carlsbad, USA). Plasmid pcDNA3.1-FLAG-α-4 giardin was cotransfected with pCMV-N-Myc into HEK293T cells and served as a negative control. At 48 h after transfection, the cells were resuspended in lysis buffer, and the lysate was used for coimmunoprecipitation assays employing the anti-FLAG mouse monoclonal antibody (CWBIO, Beijing, China) and Protein A/G Plus Agarose Immunoprecipitation Kit according to the manufacturer’s protocol (Sangon, Shanghai, China). The eluted immunoprecipitates and total cell lysates were boiled in SDS loading buffer for 10 min and then subjected to immunoblot analysis using anti-FLAG antibody and anti-Myc antibody (CWBIO, Beijing, China).

For endogenous co-immunoprecipitations, lysates from *G. lamblia* trophozoites were immunoprecipitated with prepared anti-α-4 giardin antibody as described above, and analyzed by Western blotting with anti-gEB1 antibody.

### Immunofluorescence Assays

Trophozoites grown on coverslips were fixed with 4% paraformaldehyde (PFA) at room temperature for 30 min, permeabilized with 0.5% Triton X-100 in PBS (PBS-Triton) for 30 min and incubated with 5% goat serum for 1 h at room temperature. Trophozoites were incubated with mouse monoclonal antibodies against α-4 giardin (1:100) and rabbit polyclonal anti-gEB1 antibodies (1:100), overnight at 4 °C, followed by incubation with Alexa Fluor 488-conjugated goat anti-mouse and RBITC-conjugated goat anti-rabbit secondary antibodies (1:200) (Sangon, Shanghai, China), respectively, for 1 h at room temperature. DAPI was used to stain the cell nuclei at a concentration of 5 ug/mL for 20 min at room temperature. All preparations were preserved using the anti-fade mounting media (Sangon, Shanghai, China) and examined using Laser Scanning Confocal Microscopy Leica SP8 STED 3X system.

### Statistical Analysis

All quantified results were presented as mean ± SD. The data analysis was evaluated using GraphPad Prism 8.01 statistical analysis software (GraphPad Software). Statistical comparisons among groups were performed by one-way ANOVA. P < 0.05 was regarded as significant.

## Results

### Down-Regulation α-4 Giardin for Inhibiting Proliferation and Decreasing Adherence

**Fig. 1:**
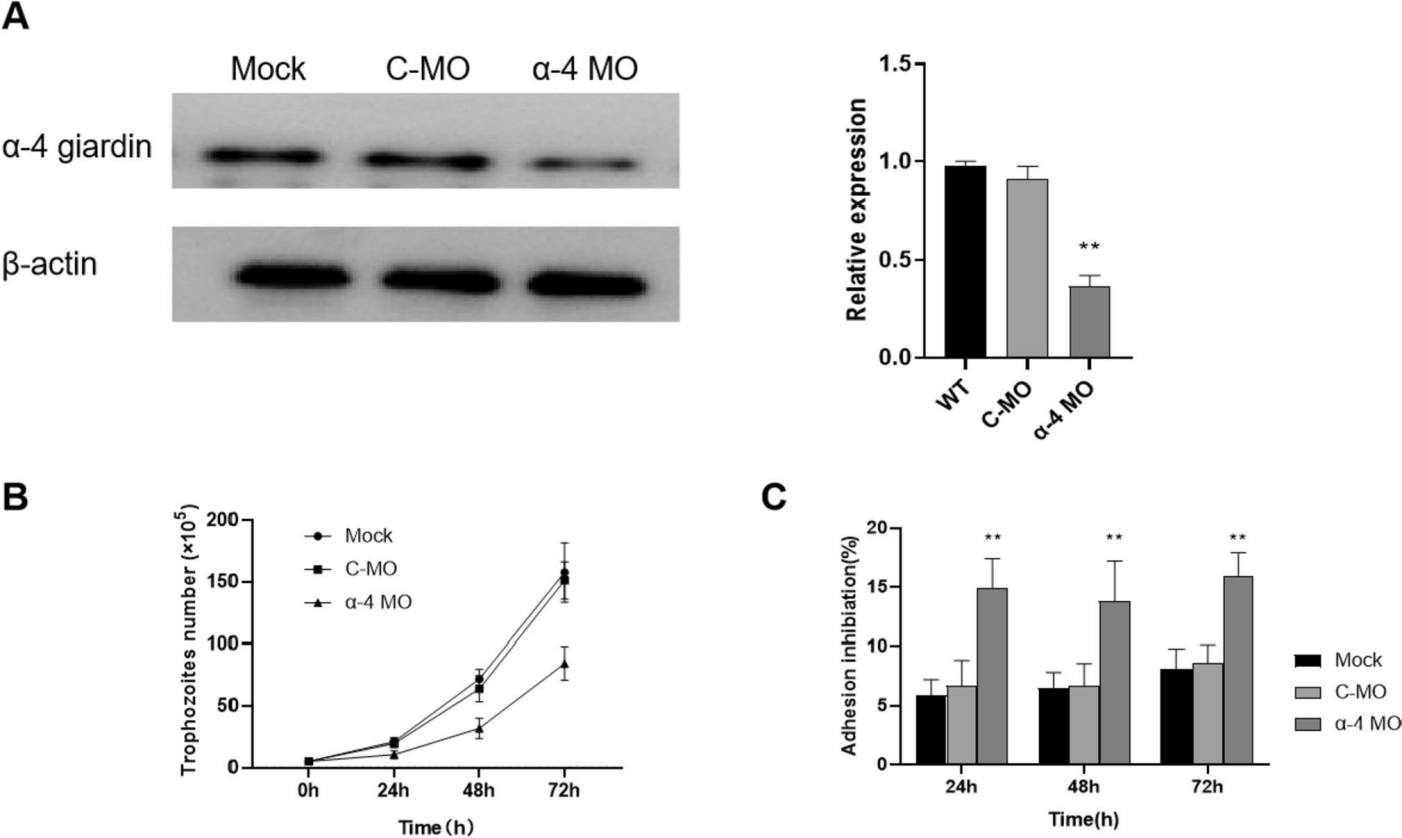
Effect of α-4 giardin knockdown on *G. lamblia* trophozoites. **A** Western blot analysis of α-4 giardin knockdown efficiency.in *G. lamblia* trophozoites. Alpha 4-giardin protein levels were measured using immunoblot assay with antibody anti-α-4 giardin. Beta-actin expression was determined as a loading control for each sample. The bands were quantified and presented as the mean ± SD of three independent experiments (right). Subsequent quantitative analysis revealed that the protein expression level of the α-4 giardin was significantly reduced following morpholino knockdown (**P < 0.01). **B** Growth rate of *G. lamblia* trophozoites transfected with α-4 MO is slower than that of C-MO and mock groups at 24 h, 48 h and 72 h. The results represent the mean of triplicate determinations ± SD of a representative experiment (*P* < 0.01). **C** Adhension inhibition rate of *G. lamblia* trophozoites transfected with α-4 MO is significant higher than that of C-MO and mock groups at 24 h, 48 h and 72 h. The results represent the mean of triplicate determinations ± SD of a representative experiment (**P < 0.01)

Morpholinos were introduced into *G. lamblia* trophozoites by electroporation and then differences were observed between the three types of cells, namely, α-4 morpholino (α-4 MO) introduced, mispair control morpholino (C-MO) introduced and sterile water treated (mock). The ability of morpholino to knock down α-4 giardin was demonstrated by western blot at 24 h after electroporation. The presence of α-4 MO had no observable effect on β-actin expression compared to control. α-4 Giardin in the mock group and C-MO group were expressed at high levels; however, it had decreased by ~ 63% in the cultures treated with 100 μM α-4 morpholino (Fig. 1).

Cell counting were performed using a Neubauer counting chamber to investigate the effects of α-4 giardin knockdown on cellular proliferation and adhesion in *G. lamblia* trophozoites. The results indicated that downregulation of α-4 giardin can significantly inhibit the proliferation and adhesion of *G. lamblia* trophozoites at each timepoint (Fig. 1B). The non-adherence of α-4 MO group was increased to ~ 15% after 24 h of electroporation (Fig. 1C), but the maximal adherence inhibition for α-4 MO group was observed at 72 h (~ 16% inhibition), which was significantly higher than mock and C-MO groups (~ 8% and ~ 8.6% inhibition, respectively).

### Yeast Two-Hybrid Screen

**Fig. 2:**
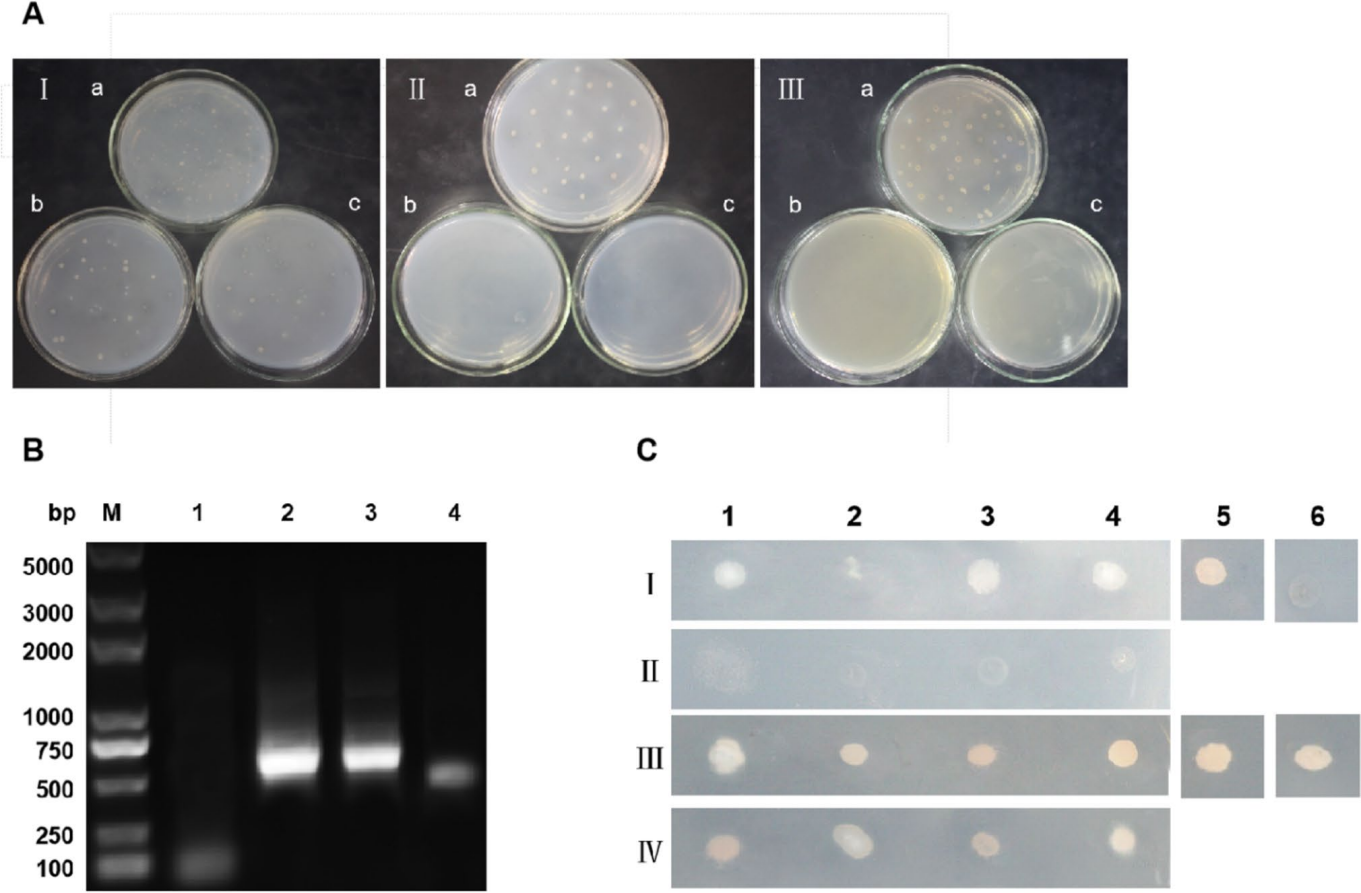
Yeast two-hybrid screen for proteins interacting with α-4 giardin. **A** Auto-activation activity of pGBKT7-α-4 in yeast cells. (I) Y187 cells transformed with pGBKT7-53. (II) Y187 cells transformed with pGBKT7-α-4. (III) Y187 cells transformed with pGBKT7. a, SD/-Trp agar medium; b, SD/-Trp/-His agar medium; c, SD/-Trp/-Ade agar medium. **B** PCR products of positive colonies from the two-hybrid cDNA library. Analysis was performed by 1% agarose gel electrophoresis. M: DNA marker; 1–4: clone 1–4. **C** Cotransformation analysis of putatively positive plasmids. I, Y187 cotransformed with pGBKT7-α-4 and each of the 4 putatively positive plasmids (1–4) grew on SD/–Leu/–Trp/–His/-Ade plates; 5: positive control (pGBKT7/p53 + pGADT7/T transformant); 6: negative control (pGBKT7 + pGADT7 transformant). II, Y187 cotransformed with pGBKT7 and each of the 4 putatively positive plasmids grew on SD/–Leu/–Trp/–His/-Ade plates. III, Y187 cotransformed with pGBKT7-α-4 and each of the 4 putatively positive plasmids (1–4) grew on SD/–Leu/–Trp plates; 5: positive control; 6: negative control. IV, Y187 cotransformed with pGBKT7 and each of the 4 putatively positive plasmids grew on SD/–Leu/–Trp plates

A yeast two-hybrid cDNA library from *G. lamblia* strain C2 trophozoites was previously constructed by our laboratory and screened for interaction partners of α-4 giardin by a yeast two-hybrid system. A bait plasmid containing coding region of α-4 giardin was constructed, and indicated that the bait protein had no toxicity in the yeast strain Y187 (Fig. 2A).

To screen for proteins interacting with α-4 giardin, Y187 cells harboring the pGBKT7-α-4 plasmid were used to mate with the constructed yeast two-hybrid cDNA library from *G. lamblia* strain C2 trophozoites according to the manufacturer’s instructions. A highly selective medium was used to screen for interacting proteins. After mating and growth on selection SD/-Trp, SD/-Leu, and SD/-Trp/-Leu media, 4 colonies were cultured on SD/–Leu/–Trp and SD/–Leu/–Trp/–His/-Ade plates, with positive ones selected on SD/–Leu/–Trp/–His/-Ade plates three times. Finally, the selected colonies were cultured in selective liquid medium (SD/-Trp/-Leu/-His/-Ade). Specific inserts for various putative positive clones were amplified by PCR and analyzed by gel electrophoresis. All 4 positive colonies were selected for culture in selective liquid medium (SD/–Leu/–Trp/–His/-Ade/kanamycin) (Fig. 2B). Specific inserts for various putative positive clones were extracted and transformed into *E. coli.* To eliminate false positives and retest interaction specificity, each of the 4 prey plasmids (pGADT7-X) was co-transformed with pGBKT7-α-4 into the yeast strain Y187, and co-transformants were assessed on SD/–Leu/–Trp/–His/-Ade plates. Of 4 prey putative positive plasmids, clones 1, 3, and 4 could grow on both SD/–Leu/–Trp and SD/-Leu/-Trp/-His/-Ade defective plates, suggesting that there may be interactions between these three clones and α-4 giardin (Fig. 2C).

Sequences of the 3 putative positive genes were analyzed for similarity using the BLAST program at NCBI (http://www.ncbi.nlm.nih.gov/BLAST/) against the *G. lamblia* genome sequence. One of the sequences had no meaningful results. The other two putative interacting proteins were *G. lamblia* end binding protein 1 (gEB1) and ribosomal protein L21.

### Interaction Between α-4 Giardin with gEB1

**Fig. 3:**
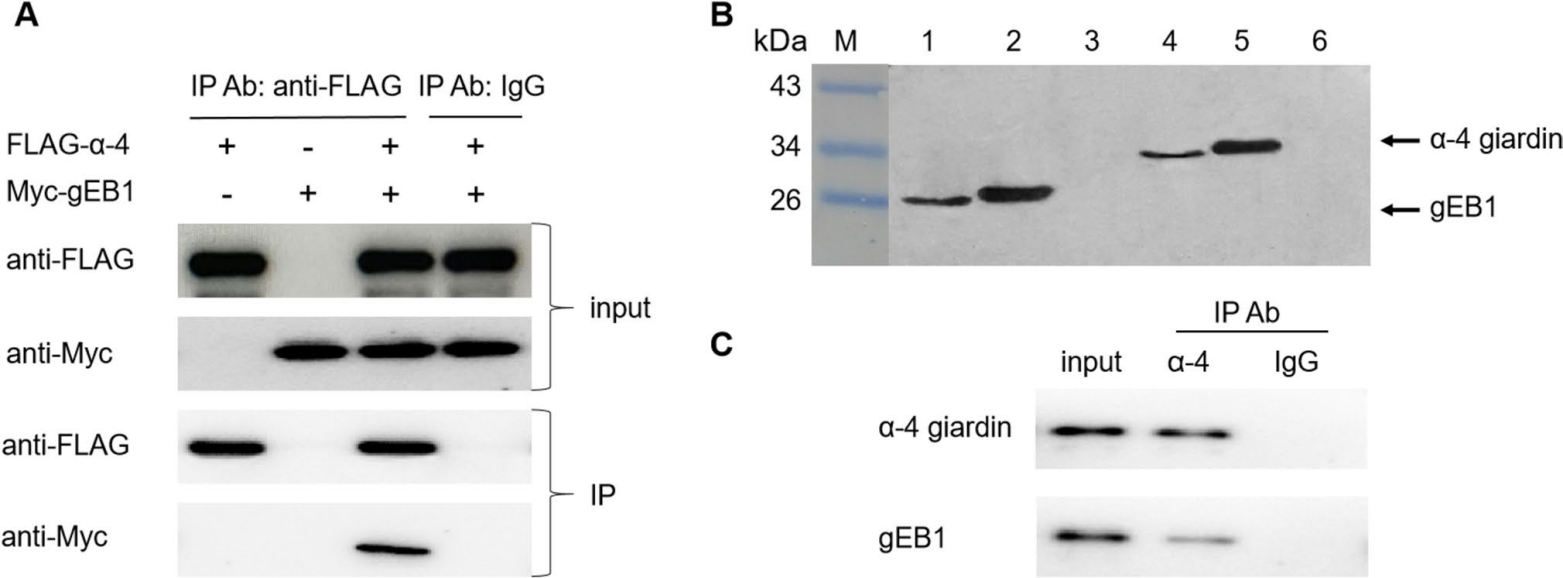
Interaction between α-4 giardin with gEB1. **A** Immunoprecipitation using HEK293T cells transfected with the indicated combination of pcDNA3.1-FLAG-α-4 giardin and pCMV-Myc-gEB1. Cell extracts were immunoprecipitated from the transfected HEK293 cells using anti-FLAG antibody and the immunoprecipitated materials were analyzed by anti-Myc antibody. Rabbit and mouse IgG antibodies were used as negative controls in the immunoprecipitation reaction. **B** Identification of antibodies specificity by Western Blot analysis. M: protein molecular marker; Lane 1: lysate of *E. coli* Rosetta (DE3) harboring the plasmid pET-28a-gEB1 without addition of IPTG was immunodetected using the anti-gEB1 (1:1000) polyclonal antibody; 2: lysate of *E. coli* Rosetta (DE3) harboring the plasmid pET-28a-gEB1 after induction by 1 mM IPTG was immunodetected using the anti-gEB1 (1:1000) polyclonal antibody; Lane 3: lysate of *G. lamblia* trophozoites was immunodetected using the anti-gEB1 (1:1000) polyclonal antibody; Lane 4: lysate of *E. coli* Rosetta (DE3) harboring the plasmid pET-28a-α-4 without addition of IPTG was immunodetected using the anti-α-4 giardin monoclonal antibody (1:1000); Lane 5: lysate of *E. coli* Rosetta (DE3) harboring the plasmid pET-28a-α-4 after induction by 1 mM IPTG was immunodetected using the anti-α-4 giardin monoclonal antibody (1:1000); Lane 6: *G. lamblia* trophozoites lysates was immunodetected using the anti-α-4 giardin (1:1000) monoclonal antibody. **C** Immunoprecipitation of endogenous α-4 giardin and gEB1 in *G. lamblia* trophozoites. Total proteins were precipitated with α-4 giardin or control IgG antibody, and detected with the anti-gEB1 antibody. Input indicates the protein expression of α-4 giardin and gEB1. Reciprocally, total proteins were precipitated with either anti-α-4 giardin or non-immune mouse IgG, and probed with anti-gEB1 antibody

To verify the interaction between α-4 giardin and gEB1, HEK293T cells were transiently transfected with pcDNA3.1-FLAG-α-4 and pCMV-Myc-gEB1 plasmids and subjected to immunoprecipitation. The results showed that FLAG-α-4 giardin and Myc-gEB1 recombinant proteins were expressed at high levels and α-4 giardin were co-immunoprecipitated with anti-FLAG antibody and immunoblotted with anti-FLAG and anti-Myc antibody, indicating that α-4 giardin interacted with gEB1 (Fig. 3A).

To perform endogenous immunoprecipitation assay, anti-α-4 giardin monoclonal antibody and anti-gEB1 polyclonal antibody were produced using synthetic peptides derived from α-4 giardin and gEB1, respectively. Western blot analysis using the lysate of trophozoites showed that the antibodies had a good specificity (Fig. 3B). The endogenous immunoprecipitation assay verified that α-4 giardin interacted with gEB1 in *G. lamblia* trophozoites (Fig. 3C).

### Identification and Immunolocalization of α-4 Giardin and gEB1

**Fig. 4:**
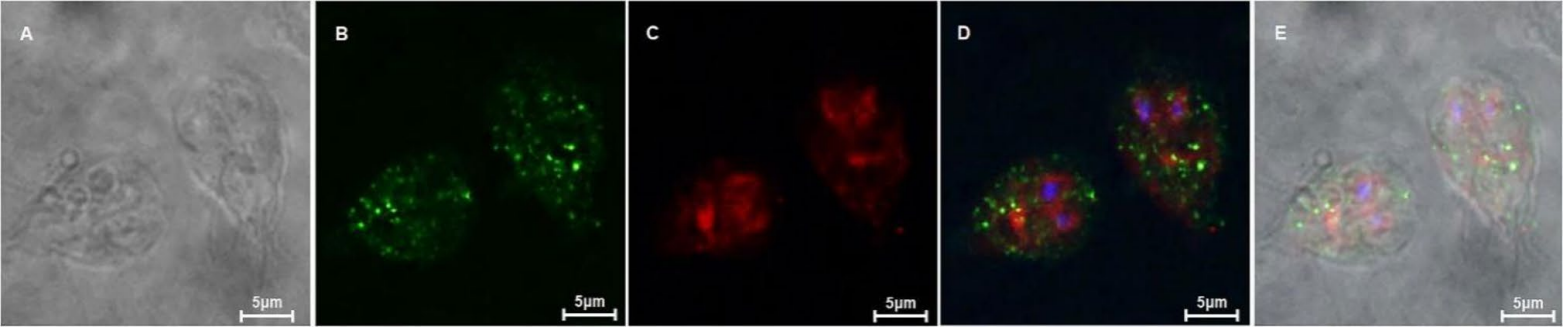
Immunolocalization of the α-4 giardin and gEB1 protein in *G. lamblia* trophozoites. **A** phase-contrast image; **B** anti-α-4 giardin fluorescence image; **C** anti-gEB1 fluorescence image; **D** merged (nuclear stained with DAPI; E. phase-contrast image merged with anti-α-4 giardin and anti-gEB1 fluorescence (nuclear stained with DAPI). White bar indicates size of 5 μm

Althoughα-4 giardin and gEB1 physically interact, it was important to verify that these two proteins are normally expressed in a manner that provides the opportunity to interact in trophozoites. In situ localizations of α-4 giardin and gEB1 were identified by immunofluorescence analysis using anti-α-4 giardin monoclonal antibody and anti-gEB1 polyclonal antibody. In trophozoites, α-4 giardin showed a scattered distribution in the in the cytoplasm. In addition, gEB1 was predominantly expressed in the nuclear membrane and nearby cytoplasm, as well as median body. A similar location was determined (Fig. 4).

## Discussion

Alpha giardin is the most studied type of giardin by far. Phylogenetic analyses showed that it belongs to homologous analogues of annexin [4], which are Ca^2+^-dependent phospholipid-binding proteins. Annexin is an intracellular protein, and accounts for about 2% of the total protein in the cell. It exists in the cell or binds to the cell membrane or cytoskeleton. Annexin is widely found in various eukaryotic cells. To date, thousands of members of the annexin family have been reported in a variety of biological species. Phylogenetic analyses showed that the annexin family is highly conserved in eukaryotes, with all having a highly conserved central domain, which can reversibly bind to phospholipids in a calcium dependent manner. According to their structures, evolutionary relationships, and locations on the chromosome, annexins can be classified into five families, including A (vertebrates), B (invertebrates), C (fungi and some groups of unicellular eukaryotes), D (plants), and E (protists). Annexins interact with various cell-membrane components involved in the structural organization of the cell, intracellular signaling by enzyme modulation and ion flux, and growth control; they also act as atypical calcium channels and exert many other important cellular physiological effects. However, functional differences among the various members of the annexin family remain puzzling.

Most eukaryotic species have 1–20 annexin genes. For example, humans have 12 annexins; *Arabidopsis thaliana* has 8, and only 2 ~ 4 are found in *Saccharomyces cerevisiae* and *Caenorhabditis elegans* [6]. *Trichomonas vaginalis*, another protozoa, has only one annexin analogue. No annexin analogues have been found in *Plasmodium* spp. and *Trypanosoma* spp. However, it is interesting that *G. lamblia*, a representative of lower eukaryotes, has up to 21 annexin analogues, including α giardin. Giardin belongs to category E annexins, with less functional studies. The α giardin family members have some sequence similarities, but are not identical. Previous studies have shown that 21 giardin variants are located in different parts of *G. lamblia* trophozoites. For example, α-3, α-5 and α-17 giardin proteins are mainly distributed in the ventral adhesive disc, while α-18 and α-20 giardin are mainly located in flagella and the cytoplasm [22]; α-8 giardin is mainly located in the cytoplasmic membrane and flagella [23]. α-4 Giardin was mainly found in the cytoplasm and the flagellar root in a previous study by our team. Different subcellular localizations suggest that there may be functional differences for α giardin family members. Assessing why *G. lamblia*, a primitive eukaryote, has so many annexins, and the functional differences among them is very important in understanding the mechanisms of action of annexins as well as the survival mechanism of *G. lamblia*. Currently, the specific functions of various α giardin proteins are mostly unknown. In this study, a vivo morpholino was employed to block the expression of α-4 giardin in trophozoites. Morpholino antisense oligonucleotides are common used to transient knockdown of gene expression by blocking translation of a targeted protein, which have been proved to be a simple and effective gene knockdown technique in *G. lamblia* research. The results showed that downregulation of α-4 giardin could impair the proliferation and adhesion of *G. lamblia* trophozoites. However, in previous study, downregulation of α-8 giardin also inhibited the proliferation of *G. lamblia* trophozoites. These results implied that knockdown experiments cannot reflect subtle functional differences among α giardin family members.

Identification of interacting proteins may be an effective approach to detect differences in the mechanisms of action among α giardin family members. Therefore, a yeast two-hybrid library containing all cDNAs of *G. lamblia* strain C2, with a capacity reaching 2.715 × 10^7^ CFU, was previously constructed by our laboratory and screened by α-4 giardin bait plasmid in this study. After screening, only two α-4 giardin interacting proteins, EB1 and ribosomal protein L21, were obtained. EB1 is a highly conserved protein in eukaryotes; its N-terminal region has a typical actin binding domain referred to as the CH domain, whereas the EB1 domain at the C-terminal end plays a role in dimerization and binding to EB1-interacting proteins. Under normal conditions, EB1 preferentially binds to fast growing microtubule ends to participate in cell cycle control. It mainly controls the dynamics of the microtubule system and promotes the proper positioning of spindles, which play a very important role in the process of mitosis [24, 25]. Knockout of the EB1 protein may result in significantly reduced mitosis in *G. lamblia* trophozoites [26, 27]. It was previously shown that actin, β-giardin and γ-giardin are also interacting proteins of EB1 [28].

To determine the interaction between α-4 giardin and gEB1 protein, co-immunoprecipitation assay was performed. First, the assay was performed by transfection of exogenous alpha-4 giardin and gEB1. However, common eukaryotic expression vectors are not suitable for gene expression in *G. lamblia* trophozoites because of the distinctive promoter structure of *G. lamblia* genes. There was no appropriate expression vector for *G. lamblia* trophozoites in our laboratory. Considering that eukaryotes have similar protein processing and transport modes, alpha-4 giardin and gEB1 genes were transfected into HEK293T cells through common eukaryotic expression vectors. Furthermore, an endogenous co-immunoprecipitation assay was also performed in *G. lamblia* trophozoites, which showed the endogenous interaction between α-4 giardin and gEB1. As a result, the interaction between the two proteins was confirmed.

Although α-4 giardin and gEB1 physically interact, it was important to verify that these two proteins are normally expressed in a manner that provides the opportunity to interact in trophozoites. For this aim, specific antibodies against the two proteins were prepared separately and the interaction of α-4 giardin and gEB1 in vivo was further confirmed by the immunofluorescence assay in *G. lamblia* trophozoites. As shown in the results, there is a partial overlap in the localisation of the two proteins, but not a complete overlap. It is speculated that the interaction between the two proteins may be temporary. Another possibility is that α-4 giardin is only one of many interacting proteins in gEB1, so the localization of α-4 giardin and gEB1 is, therefore, not completely overlapping. Anyway, the immunofluorescence assay has shown the existence of co-localization sites between α-4 giardin and gEB1, which is sufficient to demonstrate the possibility of interaction between the two proteins in vivo.

Ribosomal protein L21 is a constitutive protein of the large ribosomal subunit, and found in the cytoplasm of human cells, participating in embryogenesis [29, 30], odontogenesis [31], and the formation of age-related cataracts [32] in humans. However, no studies have confirmed direct interactions between this protein and cytoskeletal proteins. Whether an actual interaction exists between ribosomal protein L21 and α-4 giardin needs further investigation.

## Conclusion

Our results indicated that α-4 giardin was involved in proliferation and adhesion in *G. lamblia* trophozoites, and EB1, a crucial roles in mitosis, was an interacting partner of α-4 giardin. Our work lays the foundation for further investigation of functional mechanism of α-4 giardin.

## References

[1] Cama VA, Mathison BA (2015). Infections by *Intestinal Coccidia* and *Giardia duodenalis*. Clin Lab Med.

[2] Einarsson E, Ma'ayeh S, Svärd SG (2016). An up-date on *Giardia* and giardiasis. Curr Opin Microbiol.

[3] Adam RD (2001). Biology of *Giardia lamblia*. Clin Microbiol Rev.

[4] Corrêa G, Morgado-Diaz JA, Benchimol M (2004). Centrin in *Giardia lamblia* - ultrastructural localization. FEMS Microbiol Lett.

[5] Dawson SC, House SA (2010). Imaging and analysis of the microtubule cytoskeleton in *giardia*. Methods Cell Biol.

[6] Nosala C, Dawson SC (2015). The critical role of the cytoskeleton in the PAthogenesis of *Giardia*. Curr Clin Microbiol Rep.

[7] Tůmová P, Kulda J, Nohýnková E (2007). Cell division of *Giardia intestinalis*: assembly and disassembly of the adhesive disc, and the cytokinesis. Cell Motil Cytoskeleton.

[8] Katelaris PH, Naeem A, Farthing MJ (1995). Attachment of *Giardia lamblia* trophozoites to a cultured human intestinal cell line. Gut.

[9] Cruz A, Isaura Sousa M, Azeredo Z, Carolina Silva M, Figueiredo de Sousa JC, Manso O, Cabral M (2003). Comparison between two common methods for measuring *Giardia lamblia* susceptibility to antiparasitic drugs in vitro. Acta Trop.

[10] Cruz A, Sousa MI, Azeredo Z, Leite E, Figueiredo de Sousa JC, Cabral M (2003). Isolation, excystation and axenization of *Giardia lamblia* isolates: in vitro susceptibility to metronidazole and albendazole. J Antimicrob Chemother.

[11] Crossley R, Holberton DV (1983). Selective extraction with Sarkosyl and repolymerization in vitro of cytoskeleton proteins from *Giardia*. J Cell Sci.

[12] Holberton DV, Ward AP (1981). Isolation of the cytoskeleton from Giardia. Tubulin and a low-molecular-weight protein associated with microribbon structures. J Cell Sci.

[13] Morgan RO, Fernández MP (1995). Molecular phylogeny of annexins and identification of a primitive homologue in *Giardia lamblia*. Mol Biol Evol.

[14] Morgan RO, Pilar FM (1997). Distinct annexin subfamilies in plants and protists diverged prior to animal annexins and from a common ancestor. J Mol Evol.

[15] Palm JE, Weiland ME, Griffiths WJ, Ljungström I, Svärd SG (2003). Identification of immunoreactive proteins during acute human giardiasis. J Infect Dis.

[16] Weiland ME, McArthur AG, Morrison HG, Sogin ML, Svärd SG (2005). Annexin-like alpha giardins: a new cytoskeletal gene family in *Giardia lamblia*. Int J Parasitol.

[17] Wang Y, Wang Y, Yang WS, Li J, Yu Y, Shen HE, Tian XF (2012). Preparation of a polyclonal antibody specific for α-4 giardin and its immunoelectron microscopic localization. J Pathogen Biol.

[18] Lu SQ, Wang ZY, Yan G, Chen PH, Zhu H, Gao ZZ, Wang FY (1996). Four isolates of *Giardia lamblia* cultivated axenically in China and the restriction endonuclease analysis of their DNA. J Parasitol.

[19] Keister DB (1983). Axenic culture of *Giardia lamblia* in TYI-S-33 medium supplemented with bile. Trans R Soc Trop Med Hyg.

[20] Wang AL, Sepp T, Wang CC (1995). Electroporation in *Giardia lamblia*. Methods Mol Biol.

[21] Crouch AA, Seow WK, Whitman LM, Smith SE, Thong YH (1991). Inhibition of adherence of *Giardia intestinalis* by human neutrophils and monocytes. Trans R Soc Trop Med Hyg.

[22] Wu S, Pan W, Shi X, Abdullahi AY, Wang Z, Yu X, Jiang B, Li K, Xu C, Li G (2016). Immunolocalization of α18- and α12-giardin in Giardia lamblia trophozoites. Parasitol Res.

[23] Wei CJ, Tian XF, Adam RD, Lu SQ (2010). *Giardia lamblia*: intracellular localization of alpha8-giardin. Exp Parasitol.

[24] Yu X, Abdullahi AY, Wu S, Pan W, Shi X, Hu W, Tan L, Li K, Wang Z, Li G (2017). Prokaryotic expression of α-13 giardin gene and its intracellular localization in *Giardia lamblia*. Biomed Res Int.

[25] Coquelle FM, Vitre B, Arnal I (2009). Structural basis of EB1 effects on microtubule dynamics. Biochem Soc Trans.

[26] Tirnauer JS, Bierer BE (2000). EB1 proteins regulate microtubule dynamics, cell polarity, and chromosome stability. J Cell Biol.

[27] Kim J, Sim S, Kim J, Song K, Yong TS, Park SJ (2008). *Giardia lamblia* EB1 is a functional homolog of yeast Bim1p that binds to microtubules. Parasitol Int.

[28] Kang K, Kim J, Yong TS, Park SJ (2010). Identification of end-binding 1 (EB1) interacting proteins in *Giardia lamblia*. Parasitol Res.

[29] Bhavsar RB, Makley LN, Tsonis PA (2010). The other lives of ribosomal proteins. Hum Genomics.

[30] Loreni F, Francesconi A, Jappelli R, Amaldi F (1992). Analysis of mRNAs under translational control during *Xenopus* embryogenesis: isolation of new ribosomal protein clones. Nucleic Acids Res.

[31] Xie M, Kobayashi I, Kiyoshima T, Nagata K, Ookuma Y, Fujiwara H, Sakai H (2009). In situ expression of ribosomal protein L21 in developing tooth germ of the mouse lower first molar. J Mol Histol.

[32] Zhang W, Hawse J, Huang Q, Sheets N, Miller KM, Horwitz J, Kantorow M (2002). Decreased expression of ribosomal proteins in human age-related cataract. Invest Ophthalmol Vis Sci.

